# Coupled Transport and Reaction Modeling of Sorbent Particle Size Effects in Nonisothermal Packed-Bed CO_2_ Adsorption

**DOI:** 10.1021/acsomega.5c03466

**Published:** 2025-08-06

**Authors:** Joseph Amponsah, Archibong Archibong-Eso, Yesuenyeagbe Fiagbe, David O. A. Opoku, Anthony Apatika, Emmanuel Adorkor, Samuel Adjei, Ukpabio Ekpenyong

**Affiliations:** † Mechanical Engineering, 1177 Iowa State University, 2025 Black Engineering, Ames, Iowa 50011, United States; ‡ Department of Mechanical Engineering, 520132 University of Birmingham, Dubai, P.O. Box 341799, United Arab Emirates; § 98763 Kwame Nkrumah University of Science and Technology, Ashanti Region, Kumasi 0598, Ghana; ∥ Civil Engineering Department, 215749 Takoradi Technical University, Western Region, P.O.Box 256, Takoradi, Ghana; ⊥ Department of Computer and Electrical Engineering, University of Energy and Natural Resources, Brong Ahafo Region, P.O. Box 214, Sunyani, Ghana; # Department of Electrical and Electronic Engineering, 384934 Accra Institute of Technology, 22 Naa Korkoi Oblayoo Street, Greater Accra Region, Accra 0080, Ghana; ∇ Department of Mechanical Engineering, 233753 Cape Coast Technical University, P.O. Box DL 50, Cape Coast, Ghana; ○ Department of Mechanical Engineering, Cross River University of Technology, P.M.B. 1123, Calabar, Cross River, Nigeria

## Abstract

Recent studies have shown that solid sorbents offer a promising route for post-combustion CO_2_ capture. This potential remains uncertain because the influence of particle size on the capture efficiency and reactor performance has not been fully characterized. Here, we developed a two-dimensional CFD Eulerian–Eulerian model, validated it against experimental data, and applied it to simulate CO_2_ capture in a packed-bed reactor filled with spherical particles of 0.5, 0.8, and 1.5 mm diameter. A carbon-based sorbent impregnated with potassium carbonate (K_2_CO_3_) was chosen for this study due to its relevance in industrial CO_2_ capture. Under our baseline conditions of 10% CO_2_, 60 °C, this sorbent follows a Langmuir isotherm with a maximum capacity of about 1.4 mmol of CO_2_/g at 60 °C and achieves roughly 1.2 mmol/g uptake. Its moderate thermal conductivity of 0.25 W/m·K helps dissipate the heat released during adsorption, minimizing temperature gradients across the bed. Gas–solid interactions were modeled via a Eulerian–Eulerian framework, explicitly defining interphase forces to capture momentum exchange. We used the Syamlal–O’Brien correlation for drag. Smaller particles (0.5 mm) achieved nearly complete CO_2_ removal but produced a high pressure drop of 4.2 kPa. Larger particles of 1.5 mm reduced the pressure drop (0.9 kPa) but lowered the capture efficiency to 73%. Midsized particles of 0.8 mm struck a balance, reaching about 85% capture with a moderate pressure drop of 1.7 kPa. We observed that increasing the inlet gas flow by 20% shortened the breakthrough time to 23 min but slightly reduced the capture efficiency, indicating a trade-off between the flow rate and the performance. Because CO_2_ adsorption is exothermic of −145 kJ/mol, careful thermal management is required to maintain stable operation.

## Graphical abstract

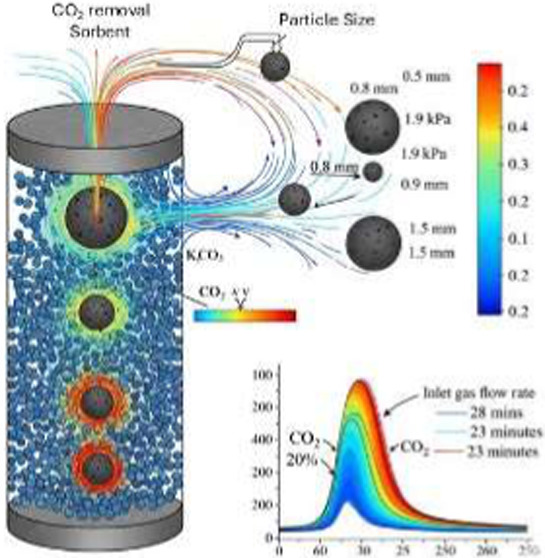

## 1 Introduction

Rising atmospheric CO_2_ levels are a major driver of climate change, prompting urgent efforts to reduce emissions. The Intergovernmental Panel on Climate Change (IPCC) warns that keeping global warming below 1.5 °C will require rapid and large-scale reductions in CO_2_ emissions. Carbon capture and storage (CCS) is a crucial technology in this context, enabling the direct removal of CO_2_ from industrial flue gases or even ambient air as part of a low-carbon strategy.^1^ Among CCS approaches, solid sorbent-based CO_2_ capture has attracted significant attention because solid sorbents can offer high selectivity and capacity for CO_2_ while potentially using less energy than traditional liquid amine scrubbing methods.^2^

The influence of particle size on the sorbent performance in CO_2_ capture systems has emerged as a critical design consideration, yet its role under nonisothermal, reactive flow conditions remains insufficiently characterized. Particularly in fixed- and fluidized-bed reactors, particle size governs not only the surface area available for gas–solid interactions but also determines the scale and dynamics of mass transfer resistance, pressure drop, and thermal transport within the bed. These interdependent factors complicate the optimization of particle size for maximizing the capture efficiency without incurring prohibitive energy penalties or system instabilities. Experimental studies have begun to delineate these trade-offs. Balsamo et al.^3^ investigated postcombustion CO_2_ capture using activated carbon and demonstrated that finer particles enhanced adsorption kinetics due to their higher surface area and better micropore accessibility. However, these benefits came at the cost of increased pressure drop, underscoring the operational constraints associated with small particle sizes in packed beds.

Santiago et al.^4^ extended this observation to supported ionic liquid phases (SILPs), where they showed that smaller SILP particles improved uptake and sorption rates due to faster diffusion and more efficient use of surface-bound ionic liquids. The study also revealed that sorption kinetics in SILPs are highly sensitive to particle-scale transport properties, reinforcing the need for size optimization in chemically complex systems. Beyond purely physical effects, several studies have highlighted how particle size mediates long-term material stability and reactivity under cyclic conditions. Wu et al.^5^ and Perejón et al.^6^ analyzed calcium looping (CaL) cycles and observed that repeated carbonation–calcination caused structural degradation and shrinkage of limestone particles, leading to a progressive loss in CO_2_ uptake capacity. Although some reactivity could be recovered through steam reactivation or thermal pretreatment, these strategies further emphasize how size and morphology evolve dynamically in operating reactors and must be accounted for in any predictive framework.

Kaya et al.^7^ investigated polyethylenimine (PEI)-functionalized silica xerogels and found that CO_2_ adsorption capacity and thermal resilience varied significantly with particle size. To capture these intertwined processes, advanced CFD modeling platforms, most notably ANSYS Fluent and CFX, have been deployed. While Fluent has been widely used for reactive packed-bed simulations in CO_2_ capture, the broader competency of ANSYS is evidenced by refs 8,9 who employed ANSYS CFX to model the hydrogen behavior and thermo-pressure dynamics scenarios. Their work demonstrated CFX’s capability to resolve detailed pressure, temperature, and concentration fields under severe accident conditions, effectively simulating gas accumulation, stratification, and safety system performance. This highlights the platform’s suitability for analogous modeling challenges in CO_2_ adsorption, where accurate resolution of coupled thermal and mass transfer is equally critical.

Increased particle size is correlated with a diminished adsorption performance, but also improved mechanical integrity, suggesting that particle growth during amine loading can both aid and hinder the performance, depending on the reactor’s thermal profile. In the context of ambient and postcombustion capture, Jiang et al.^10^ and Sher et al.^11^ studied pelletized PEI–mesocellular silica and biomass-derived activated carbons, respectively, and consistently observed that midsized particles of 0.5–1.0 mm offered an optimal balance. These particles minimized diffusion limitations while avoiding excessive pressure drop and thermal gradients, thus preserving the performance across multiple cycles. These empirical insights underscore a broader principle: particle size cannot be optimized in isolation but must be evaluated in the context of coupled transport and reaction phenomena, particularly in thermally sensitive systems where adsorption is exothermic and spatially varying temperature fields emerge.

Advanced porous materials such as metal–organic frameworks (MOFs), zeolites, and functionalized carbons exemplify solid sorbents with tunable properties to enhance CO_2_ adsorption.^9,12^ Computational modeling has become an indispensable tool for studying these materials, allowing researchers to predict the sorbent performance, understand adsorption mechanisms, and screen new sorbent candidates more efficiently than purely experimental approaches.^8,13^ However, despite modeling and material development progress, gaps remain between model predictions and the real-world performance. Many models struggle to accurately predict the sorbent behavior under practical operating conditions at industrial gas flow rates, elevated pressures, or temperature fluctuations.^14^ Bridging this gap requires models incorporating realistic process conditions and detailed sorbent characteristics.

One key practical factor influencing the solid sorbent performance is particle size. Sorbent particle size directly affects the available surface area for adsorption and the mass transfer rates of CO_2_ into the particles. In general, smaller particles expose more surface area per unit volume, which can increase adsorption capacity and kinetics. Powdered activated carbon in the micrometer range (1–100 μm) exhibits rapid CO_2_ uptake due to its high surface area.^2^ However, extremely small or fine particles (<50 μm) packed in a bed create a high flow resistance, leading to significant pressure drops that raise energy consumption for pumping the gas.^15^

Recent studies suggest that very small particles maximize capture but impede flow, while very large particles allow easy flow but capture less CO_2_.^16^ In industrial practice, engineers choose particle sizes to balance these factors. Pressure swing adsorption (PSA) systems often use sorbent pellets of about 1–3 mm diameter because this intermediate size provides a good compromise between adsorption capacity and manageable pressure drop.^17^ CFD has become a cornerstone in modern engineering analysis because it lets researchers visualize velocity, pressure, and species-transport fields that are difficult and often impossible to measure experimentally. Recent work by ref 18 combined high-resolution laser Doppler velocimetry with CFD to reproduce radial velocity profiles in a stirred tank reactor, illustrating how numerical flow models complement experiments and accelerate equipment design. Their study highlights two advantages that are equally important for the present work: (i) CFD can resolve three-dimensional flow structures inside packed or agitated domains and (ii) once validated, the model can be used to explore operating conditions that lie beyond the reach of laboratory testing.

Building on these strengths, the current investigation applies a validated CFD framework to quantify how sorbent particle size influences the CO_2_ capture efficiency and pressure drop in packed-bed contactors, providing design guidance that would be impractical to obtain by experiment alone. The effect of particle size on CO_2_ capture is generally understood, but detailed data under real operating conditions are still limited. Smaller particles tend to capture more CO_2_ because they have more surface area, but they also create more resistance to gas flow, which increases energy use. Larger particles allow the gas to flow more easily, but they capture less CO_2_ due to slower diffusion and lower surface contact. In this work, we use a detailed CFD model to study how different particle sizes of 0.5, 0.8, and 1.5 mm affect the system performance in a packed-bed reactor. All particles are tested under the same conditions so we can clearly see how size affects the capture efficiency, pressure drop, breakthrough time, and heat behavior. We also look at how the gas flow rate interacts with particle size to influence the overall performance.

The objectives of this study are 2-fold:

- Model Development: To establish a CFD-based modeling framework that captures gas-solid interactions, adsorption kinetics, and thermal effects to predict the sorbent performance under industrially relevant conditions.
- Particle Size Impact Assessment: To measure how particle size between 0.5 and 1.5 mm affects CO_2_ capture and pressure drop. This size range reflects a common practice in industrial systems, where engineers must balance the high surface area for adsorption against the increased flow resistance associated with smaller particles.^19^

## 2 Methodology

### 2.1 Adsorbent and Reactor Description

As shown in Figure 1, we consider a bench-scale packed-bed reactor for CO_2_ capture using a representative solid sorbent. The reactor is modeled as a vertical cylinder of 0.23 m in height and 0.04 m in diameter packed with spherical sorbent particles. The dimensions are comparable to laboratory CO_2_ capture columns, and the methodology can be extended to larger industrial units. The sorbent material is chosen to mimic a typical high-performance CO_2_ adsorbent. In our case, we use a carbon-based sorbent impregnated with potassium carbonate (K_2_CO_3_), which has properties representative of industrial solid sorbents. The sorbent’s CO_2_ adsorption follows a Langmuir isotherm, with a measured maximum capacity of about 1.4 mmol of CO_2_ per gram at 60 °C and around 1.2 mmol/g uptake under our baseline conditions of 10% CO_2_ at 60 °C. The sorbent’s thermal conductivity (0.25 W/m·K) contributes to effective heat dissipation during CO_2_ adsorption, which reduces the risk of significant temperature gradients in the bed. It is also mechanically stable, with only 5% mass loss after 50 adsorption–desorption cycles in tests, indicating good durability for industrial use. The combination of durability and moderate thermal conductivity makes the sorbent well suited for repeated cycling and helps manage heat buildup during operation, which aligns with the conditions modeled in the simulation.

**Figure 1:**
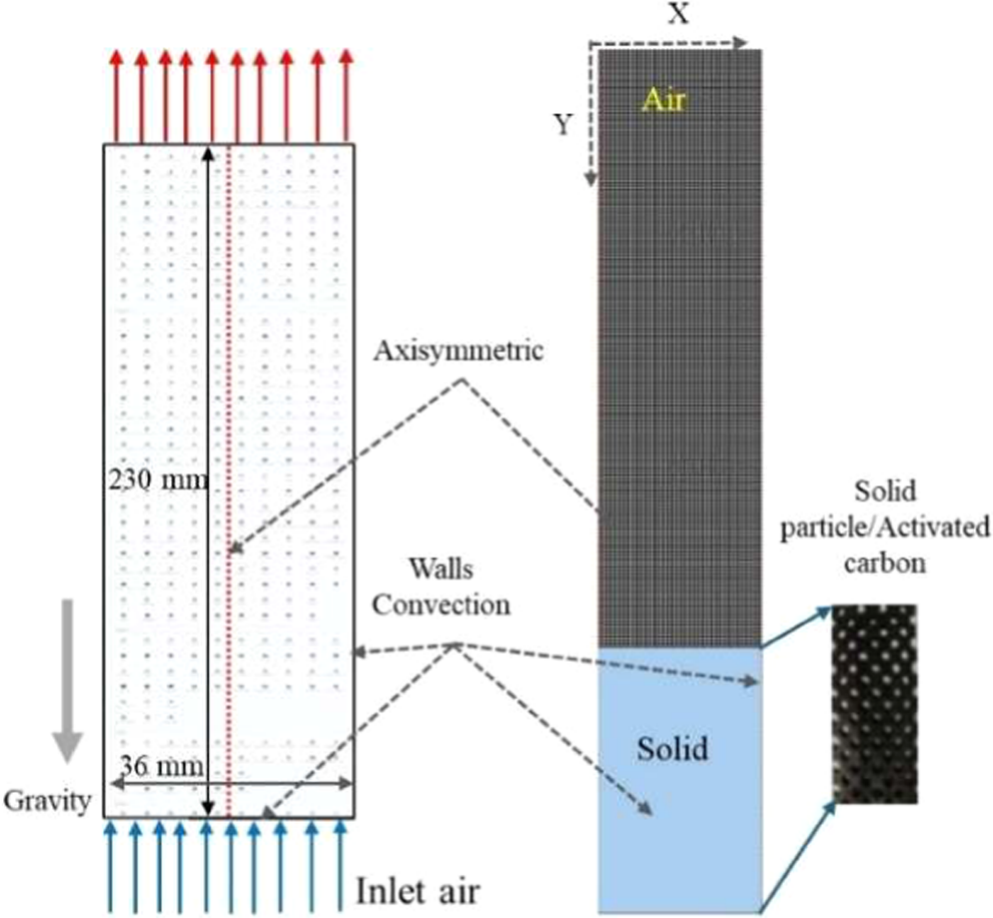
Axisymmetric reactor system with inlet air, solid particles/activated carbon, wall convection effects, and gravity direction. Geometry dimensions include a height of 230 mm and a width of 36 mm.

The CO_2_ capture process is envisioned as a cyclic adsorption–regeneration operation. During the adsorption phase, the inlet gas which mimics flue gas is passed through the packed bed at 60 °C so that the sorbent captures CO_2_. Once the sorbent nears saturation, the bed would be heated to 110–120 °C to regenerate the sorbent by releasing the captured CO_2_. Under baseline adsorption conditions, the system is designed such that the CO_2_ removal efficiency is initially high around 80–85% of incoming CO_2_ is captured, and the outlet CO_2_ starting to rise rapidly occurs after roughly 25–30 min of operation. We focus our modeling on the adsorption phase, where fluid dynamics and adsorption kinetics determine how quickly the sorbent becomes saturated and how much CO_2_ can be captured before the breakthrough.

### 2.2 Model Validation

We validated and verified our model using experimental data from Zhang et al.,^19^ who investigated the impact of particle size on CO_2_ hydrate formation within porous media, focusing on its implications for large-scale carbon capture and storage. Their study highlighted the significant role of particle size in determining gas consumption rates and pressure variations. We analyzed experimental results using a 45 μm quartz sand medium to validate the CFD model. The experimental data set used for validation consists of pressure variation curves and gas consumption rate profiles. These were compared with the CFD predictions to assess the accuracy of the numerical setup in replicating the hydrate formation behavior under controlled conditions. The experimental setup, adapted from Zhang et al.,^19^ involved a high-pressure reactor vessel with a total 300 mL volume filled with 45 μm quartz sand particles fully saturated with deionized water, as shown in Figure 2. The reactor was immersed in a temperature-controlled alcohol bath maintained at 275.15 K. CO_2_ gas with 99.99% purity was injected into the reactor at an initial pressure of 3 MPa. Table 1 summarizes key experimental parameters measured during the study, which were used as input conditions for the CFD model to replicate and validate the hydrate formation behavior.

**Figure 2:**
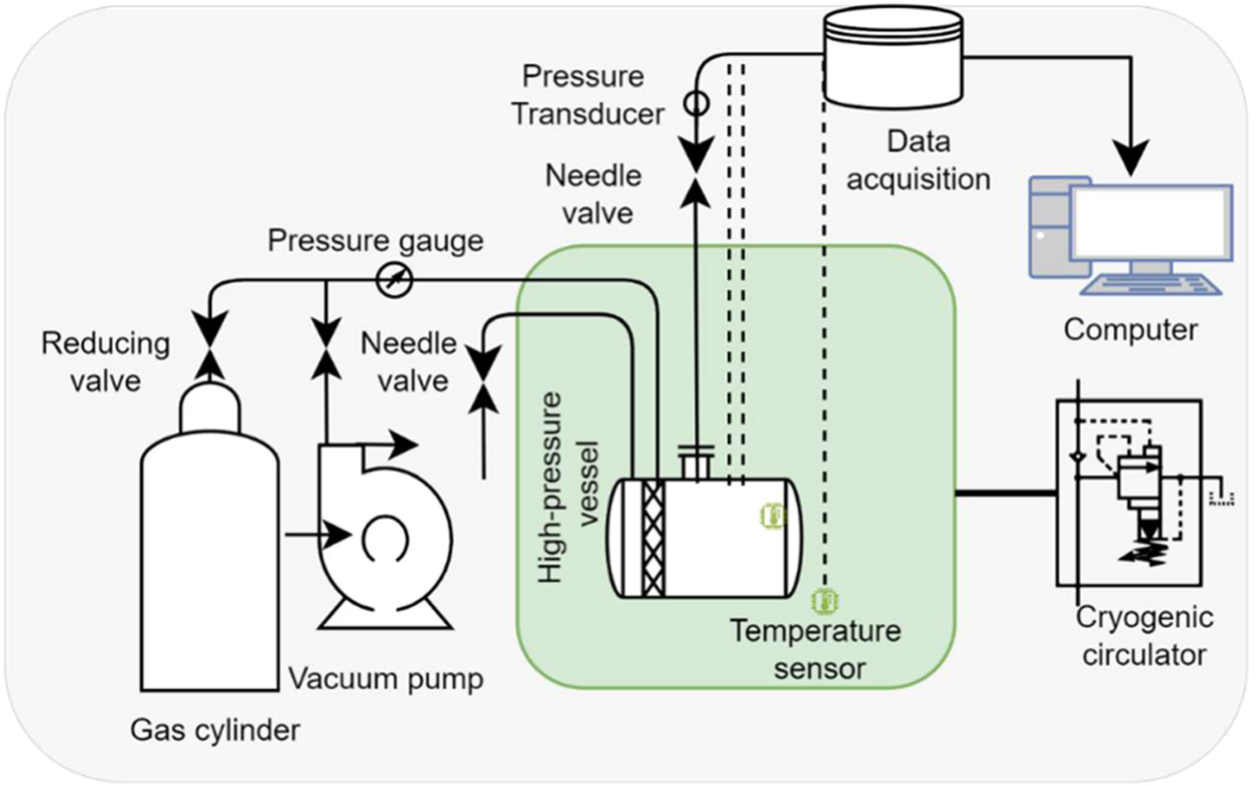
Schematic representation of the experimental setup used for CO_2_ hydrate formation in a 45 μm quartz sand porous medium. The setup includes a high-pressure reactor vessel, a thermostatically controlled alcohol bath for temperature regulation, and precision pressure and temperature sensors for continuous data acquisition. The system enables real-time gas consumption and hydrate formation monitoring under controlled conditions. Reprinted with permission from Zhang et al.,^19^ Experimental study on the influence of particle size and grain grading on the CO_2_ hydrate formation and storage process in porous media. Energy, 305, 132328 (2024). Copyright 2024 Elsevier.

**Table 1:** Experimental Data Used for CFD Validation of CO_2_ Hydrate Formation^20^

parameter	initial input
reactor volume	300 mL
particle size	45 μm
temperature	275.15 K
initial pressure	3 MPa
gas purity	99.99% CO_2_
water saturation	100%
time step interval	5 s
solver	transient (ANSYS Fluent R1)
boundary conditions	no-slip conditions at reactor walls
convergence criteria	10^–6^ residual threshold
measured outputs	pressure variation, gas consumption rate

Temperature and pressure variations were continuously monitored using high-precision sensors, recording data at 5 s intervals throughout the experiment. The hydrate formation process was identified by observing pressure declines and gas uptake rates, reflecting the kinetics of nucleation and hydrate growth. A CFD model was developed to simulate experimental conditions, ensuring that key boundary conditions, such as pressure, temperature, and water saturation, were accurately replicated. The geometry shown in Figure 1 was recreated in the computational domain, with the porous medium modeled as a packed bed with interstitial water. Hydrate formation kinetics were incorporated by coupling mass transfer-driven CO_2_ dissolution with heat release from the exothermic hydrate formation reaction. Convergence criteria were set at a 10^–6^ residual threshold, and no-slip boundary conditions were applied to the reactor walls. The validation compares the CFD results with experimental pressure variations and gas consumption data. The CFD and experimental data exhibited a characteristic initial rapid pressure drop due to CO_2_ dissolution, followed by a gradual decline as hydrate formation progressed.

The numerical model successfully captured this trend, as shown in Figure 3. Gas consumption rate profiles were also closely matched, with the CFD model effectively capturing the initial rapid increase in CO_2_ uptake. However, a slight underprediction was observed in the later stages of hydrate formation, likely due to simplifications in nucleation kinetics. These results confirm that the CFD model accurately represents the hydrate formation dynamics in a porous medium with a 45 μm particle size. The strong agreement between simulated and experimental data validates the numerical setup, reinforcing its applicability for further investigations into solid sorbent-based CO_2_ capture and optimization of hydrate formation conditions.

**Figure 3:**
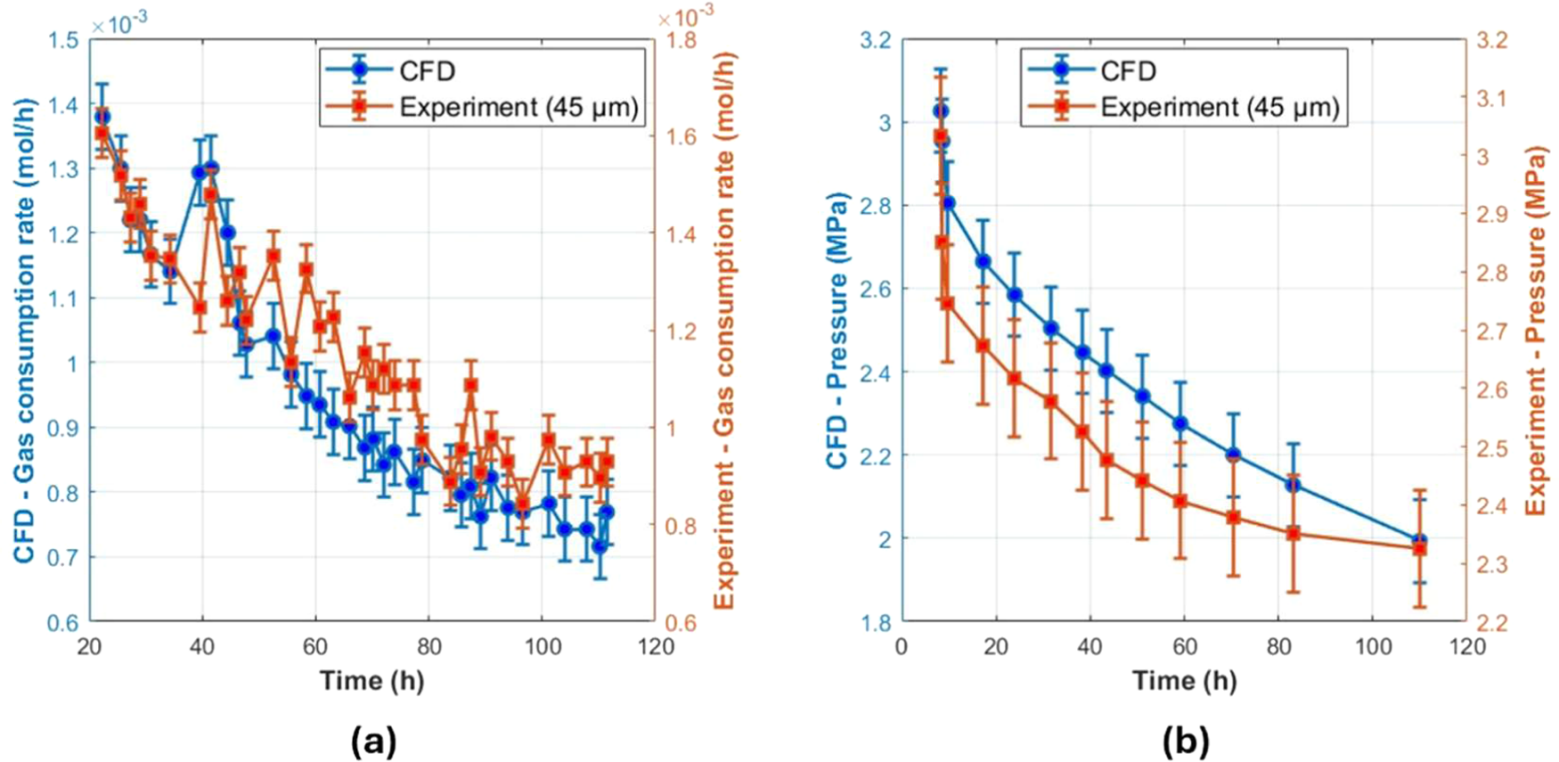
(a) Gas consumption rate over time for CO_2_ hydrate formation in a 45 μm quartz sand porous medium. CFD results are shown as hollow blue triangles, and experimental results are shown as solid brown circles. Both data sets are plotted using a unified *y*-axis for direct comparison. (b) Pressure variation during CO_2_ hydrate formation in a 45 μm quartz sand porous medium. CFD results are represented by hollow blue triangles, and experimental results are shown as solid brown circles. A single *y*-axis is used to represent the pressure in MPa.

### 2.3 Governing Equations and Multiphase Modeling

The gas–solid interactions in the CO_2_ capture process were modeled using a Eulerian–Eulerian framework, where interphase forces were explicitly defined to account for momentum exchange. The drag force was modeled using the Syamlal–O’Brien correlation, which considers the relative velocity between phases and is expressed as

1 $$F_{d}=\frac{3}{4}C_{d}\frac{\rho_{g}}{d_{p}}\left|u_{g}-u_{s}\right|\left(u_{g}-u_{s}\right)$$

where *C*_d_ is the drag coefficient, ρ_g_ is the gas density, *d*_p_ is the particle diameter, and **u**_g_ and **u**_s_ are the velocities of the gas and solid phases, respectively. No modifications were applied to this correlation. This study did not include lift forces arising from velocity gradients and induce transverse motion in the solid phase. The lift force, typically represented as

2 $$F_{l}=C_{l}\rho_{g}\left(u_{g}-u_{s}\right)\times \left(\nabla \times u_{g}\right)$$

was set to zero, assuming that transverse effects were minimal under the studied flow conditions. Similarly, turbulent dispersion forces, which account for fluctuations in turbulent regions, were not modeled explicitly, given that drag was the dominant mechanism governing phase interactions. Interphase turbulence interactions, which typically contribute to turbulent energy transfer, were neglected, as the energy dissipation between phases was assumed to be minor. Virtual mass forces, responsible for additional acceleration effects due to the relative motion of phases, were also omitted, considering that

3 $$F_{\mathrm{vm}}=C_{\mathrm{vm}}\rho_{g}V_{s}\left(\frac{D}{\mathrm{Dt}}\left(u_{g}-u_{s}\right)\right)$$

where *C*_vm_ is the virtual mass coefficient and *V*_s_ is the solid-phase volume fraction. This force was set to zero since the relative acceleration between phases was not a primary concern. The solid pressure was computed using Lun et al.’s model:

4 $$P_{s}=\rho_{s}\epsilon_{s}\Theta_{s}+2\left(1+e\right)g_{0}\rho_{s}\epsilon_{s}^{2}\Theta_{s}$$

where ρ_s_ is the solid-phase density, ϵ_*s*_ is the solid volume fraction, Θ_s_ is the granular temperature, *e* is the coefficient of restitution, and *g*_0_ is the radial distribution function, also defined following Lun et al.’s approach. Table 2 shows the key settings incorporated in the model.

**Table 2:** Summary of Model Selection

model component	selected model
drag model	Syamlal–O’Brien
lift coefficient	none
turbulent dispersion	none
virtual mass coefficient	none
granular viscosity	syamlal–O’Brien
bulk viscosity	nonstant (0)
solid pressure	Lun et al.
granular temperature	algebraic
packing limit	constant (0.63)
radial distribution	Lun et al.
elasticity modulus	constant

### 2.4 Sorbent Properties

A sorbent material with density, pore volume, and surface area values of 700 kg/m^3^, 0.35 cm^3^/g, and 650 m^2^/g, respectively, is selected for this study. The properties align with carbon-based adsorbents predominantly used for CO_2_ capture.^21^ Its thermal conductivity, measured at 0.25 W/m, facilitates effective heat dissipation and prevents significant temperature gradients within the bed during operation. The material’s adsorption characteristics follow a Langmuir isotherm, with a maximum capacity of 1.4 mmol/g at 60 °C and an actual uptake of 1.2 mmol/g under baseline conditions. The presence of moisture in the gas feed slightly reduced the adsorption capacity by approximately 0.1 mmol/g due to competitive adsorption effects.

### 2.5 Solid Sorbent Carbon Capture System

The simulation was done in semibatch mode, alternating between adsorption at 60 °C and regeneration at 110–120 °C. The CO_2_ removal efficiency averaged 80–85%, with a breakthrough occurring after 25–30 min. Early in the adsorption cycle, the top 20 cm of the bed captured most of the CO_2_ load, with the saturation zone gradually migrating downward. Energy consumption analysis indicated that the blower required 1.1–1.3 kW, while the regeneration heater added 0.8–1.0 kW, resulting in a net energy cost of 2.5 MJ/kg CO_2_.

### 2.6 Simulation Setup and Assumptions

We employed a Eulerian–Eulerian model to simulate the gas flow through the packed bed and the adsorption of CO_2_ onto the sorbent particles. A Eulerian–Eulerian two-phase approach was used, treating the gas and solid phases as interpenetrating continua. The method captures interactions between the flowing gas and the fixed, porous particle bed, including momentum exchange, mass transfer, and heat transfer. Mass conservation (continuity) for both gas and solid phases ensures that any mass transfer of the CO_2_ adsorption is accounted for by a corresponding loss in the gas and gain in the solid. The continuity equations include source terms for CO_2_ adsorption, when CO_2_ is adsorbed from the gas, a mass source term appears in the solid phase and a corresponding sink appears in the gas phase. Equations 1–4 show the energy equations for the gas and solid phases:

5 $$\frac{\partial }{\partial t}\left(\varepsilon_{s}\rho_{s}\right)+\nabla \cdot \left(\varepsilon_{s}\rho_{s}U_{s}\right)=R_{s}$$

6 $$\frac{\partial }{\partial t}\left(\varepsilon_{g}\rho_{g}\right)+\nabla \cdot \left(\varepsilon_{g}\rho_{g}U_{g}\right)=R_{g}$$

7 $$\frac{\partial }{\partial t}\left(\varepsilon_{g}\rho_{g}\right)+\nabla \cdot \left(\varepsilon_{g}\rho_{g}U_{g}\right)=R_{g}$$

8 $$\frac{\partial }{\partial t}\left(\varepsilon_{g}\rho_{g}h_{g}\right)+\nabla \cdot \left(\varepsilon_{g}\rho_{g}u_{g}h_{g}\right)=-\varepsilon_{g}\frac{\partial p}{\partial t}+\nabla \cdot \left(\varepsilon_{g}k_{g,\mathrm{eff}}\nabla T_{g}\right)+h_{\mathrm{gs}}\left(T_{s}-T_{g}\right)-{ṁ}_{\mathrm{ads}}h_{\mathrm{ads}}$$

9 $$\frac{\partial }{\partial t}\left(\varepsilon_{s}\rho_{s}h_{s}\right)+\nabla \cdot \left(\varepsilon_{s}\rho_{s}u_{s}h_{s}\right)=-\varepsilon_{s}\frac{\partial p}{\partial t}+\nabla \cdot \left(\varepsilon_{s}k_{s,\mathrm{eff}}\nabla T_{s}\right)+h_{\mathrm{gs}}\left(T_{g}-T_{s}\right)+{ṁ}_{\mathrm{ads}}h_{\mathrm{ads}}$$

Here, *h*_g_ and *h*_s_ are the specific enthalpies, and *T*_g_ and *T*_s_ are the temperatures of the gas and solid phases. The terms *k*_g,eff_ and *k*_s,eff_ are the effective thermal conductivities, and *h*_gs_ is the interphase heat transfer coefficient. The term *ṁ*_ads_ *h*_ads_ accounts for the heat effects due to adsorption.

A convection–diffusion equation tracks the CO_2_ concentration (mass fraction) in the gas, with a sink term corresponding to the adsorption rate onto the sorbent. The effective diffusivity of CO_2_ in the gas through the porous bed is set to 0.140 cm^2^/s for CO_2_ in the gas mixture. The transport of CO_2_ within the gas phase is described by

10 $$\frac{\partial }{\partial t}\left(\varepsilon_{g}\rho_{g}Y_{{\mathrm{CO}}_{2}}\right)+\nabla \cdot \left(\varepsilon_{g}\rho_{g}u_{g}Y_{{\mathrm{CO}}_{2}}\right)=\nabla \cdot \left(\varepsilon_{g}\rho_{g}D_{\mathrm{eff}}\nabla Y_{{\mathrm{CO}}_{2}}\right)-{ṁ}_{\mathrm{ads}}$$

*Y*_CO_2__ is the mass fraction of CO_2_ in the gas phase, and *D*_eff_ is the effective diffusivity. The term *ṁ*_ads_ represents the mass rate of CO_2_ adsorption onto the solid sorbent.

CO_2_ adsorption is exothermic, releasing about −145 kJ per mole of CO_2_ adsorbed. In the model, this release is treated as a source term in the energy equation for the solid phase and a corresponding heat loss from the gas. The sorbent’s thermal conductivity and the assumption of some cooling wall heat losses help maintain the bed close to isothermal. Isothermal condition assumption: for simplicity and to focus on mass transfer effects, we assume that the adsorption process operates near-isothermally at 60 °C. The assumption of near-isothermal operation at 60 °C was adopted in this study to simplify the computational framework and focus on the mass transfer dynamics governing CO_2_ adsorption.

This simplification is supported by the relatively high thermal conductivity of the selected carbon-based sorbent is 0.25 W/m·K, which promotes rapid heat dissipation, as well as by the inclusion of wall convection effects in the boundary conditions. These factors together help minimize thermal gradients within the packed bed. However, we recognize that in real-world applications, localized heat release from the exothermic adsorption process may lead to transient temperature variations, particularly under high-loading or rapid-cycle conditions. Future work should incorporate nonisothermal modeling to capture these thermal effects more accurately. Due to the sorbent properties and reactor design, the heat released during adsorption is rapidly dissipated through the bed and reactor walls, which prevents significant temperature increases. Based on this, a fixed temperature of 60 °C was applied in the adsorption simulations, with the understanding that effective temperature control would be required in a practical system to maintain such conditions. The continuity equations for the gas and solid phases ensure mass conservation within the system. They are expressed as

11 $$\frac{\partial }{\partial t}\left(\varepsilon_{s}\rho_{s}X_{s}\right)+\nabla \cdot \left(\varepsilon_{s}\rho_{s}U_{s}X_{s}\right)=\nabla \cdot \left(D_{s}\nabla X_{s}\right)+R_{s}$$

where ε_g_ and ε_s_ are the volume fractions of the gas and solid phases, respectively, satisfying:

12 $$\varepsilon_{g}+\varepsilon_{s}=1$$

The symbols *g*_o_ and ρ_s_ denote the densities, while **u**_g_ and **u**_s_ represent the velocities of the gas and solid phases. *S*_mass,g_ and *S*_mass,s_ are mass source terms arising from adsorption or desorption reactions. During CO_2_ adsorption, the mass transfer rate from the gas to the solid phase is represented by *ṁ*_ads_, leading to

13 $$S_{\mathrm{mass},g}=-{ṁ}_{\mathrm{ads}}$$

14 $$S_{\mathrm{mass},s}={ṁ}_{\mathrm{ads}}$$

The initial condition for this study is summarized in Table 3. The packed-bed porosity void fraction is about 0.40, a typical value for random packing of roughly spherical particles. The inlet gas composition is 99.9% pure CO_2_ with the balance being inert N_2_ and a small fraction of water vapor to represent humid flue gas. The total flow rate of gas into the bed is set such that the superficial velocity is 0.033 m/s; in our base case, this corresponds to approximately 1.0 kg of gas per minute flowing through the reactor.^22^ We also explored a higher flow case (1.2 kg/min, a 20% increase) to examine the effect of the gas residence time on the capture performance. The gas enters at 60 °C and atmospheric pressure. The sorbent particles are initially free of CO_2_ at the start of adsorption. As shown in Figure 4, the model geometry, boundary conditions, and grid design incorporate key physical phenomena such as mass transfer, hydrodynamics, and external forces. Air enters from the right, where it is influenced by gravity, wall convection, and drag effects, while mass transfer progresses toward the outlet on the left.

**Figure 4:**
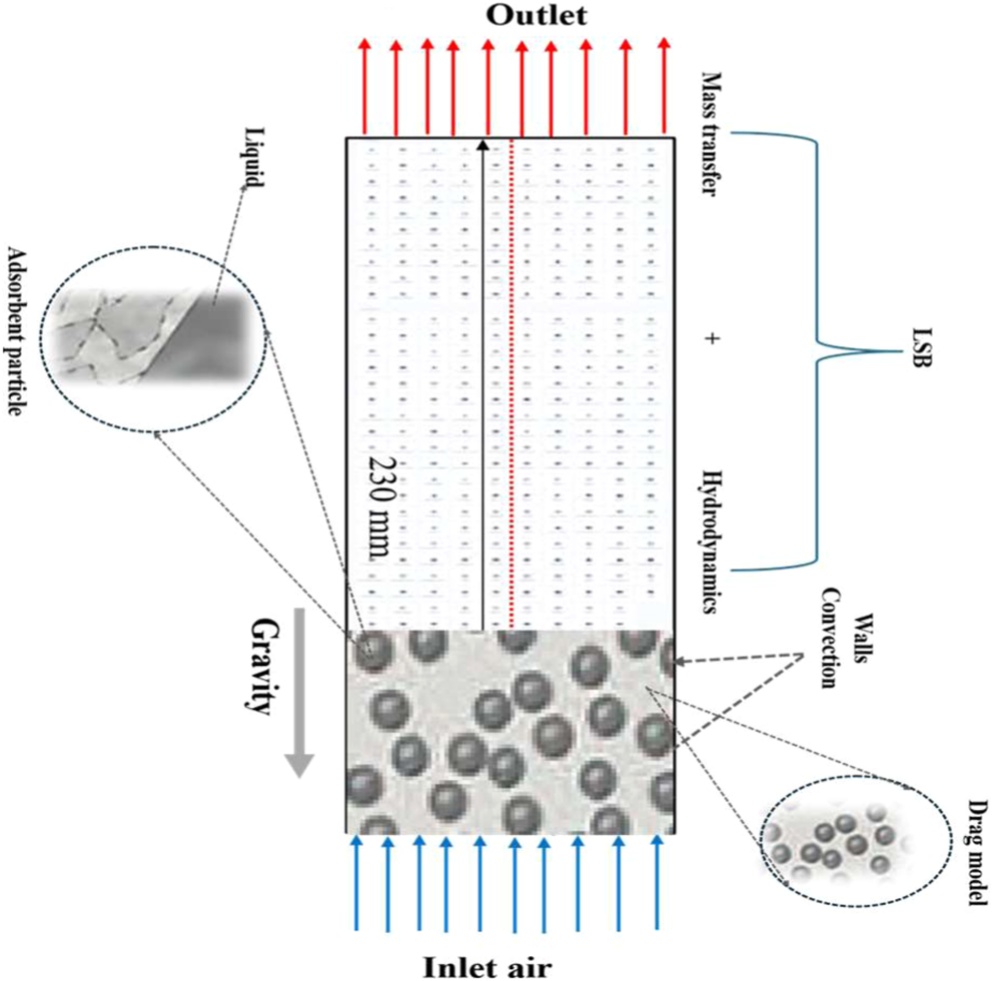
Geometry, boundary condition, and grid design of the system, including mass transfer, hydrodynamics, and relevant forces. The inlet air enters from the right, influenced by gravity, wall convection, and drag effects, while mass transfer occurs toward the outlet on the left.

**Table 3:** Initial Conditions for the CFD Model

description	value
particle diameter [mm]	0.5, 0.8, and 1.5
particle density [kg m^–3^]	1200
K_2_CO_3_ content [%]	40
reactor height [m]	0.7/6.0
reactor diameter [m]	0.040/0.030
gas diffusivity [cm^2^ s^–1^]	CO_2_ (0.140), H_2_O (0.220), N_2_ (0.210)
initial temperature [K]	360
inlet gas velocity [m s^–1^]	0.033 m/s
inlet solid flux [kg m^–2^ s^–1^]	38
inlet gas composition	CO_2_:H_2_O:N_2_ = 10:68:22
restitution coefficient	0.96
wall restitution coefficient	0.88
secularity coefficient	0.60

### 2.7 Granular Temperature Equation

The granular temperature, Θ, characterizes the kinetic energy associated with solid particles’ random, fluctuating motion, analogous to the thermal temperature in gases. It is defined as

15 $$\Theta =\frac{1}{3}\left\langle c_{s}^{\prime 2}\right\rangle$$

where ⟨*c*_*s*_^′2^⟩ is the mean square of the particle velocity fluctuations, and *c*_s_^′^ is relative to the mean particle velocity *u*_s_.

We examined three particle sizes for the sorbent: 0.5, 0.8, and 1.5 mm in diameter. These represent a small particle scenario, a medium (baseline) scenario, and a large particle scenario, respectively. The 0.8 mm size is chosen as a baseline since it is in the middle of the common range for packed-bed adsorbents (1 mm). The 0.5 mm particles illustrate the effects of using a finer, high-surface-area sorbent, whereas 1.5 mm represents a coarser sorbent that is easier to flow gas through. We compared these to identify how particle size impacts performance metrics. All other properties of the sorbent including the material, porosity, and adsorption isotherm are kept the same so that differences in the performance can be attributed to particle size alone.

As shown in Figure 5, we performed a grid-independent study to ensure the numerical accuracy of the CFD simulations. Simulations were run with mesh resolutions ranging from 200,000 to 400,000 computational cells for the 2D domain. The predicted pressure drops across the bed and the timing of CO_2_ front arrival varied by less than 3% between the medium and finest grids, which indicates that the mesh resolution was adequate for reliable results. The following results are based on the grid-refined simulations. We used a pressure-based solver with second-order upwind discretization for the convection terms to improve solution accuracy. Each simulation was run until a pseudosteady state or complete breakthrough was reached. Convergence was monitored by tracking residuals of the equations; all residuals were driven below 10^–4^, and mass balance was verified at the inlet and outlet. We also cross-checked the model against simplified one-dimensional analytical calculations of the breakthrough time and pressure drop, finding consistent trends and values. Cross-verification with available data increases confidence that the model captures the key physical mechanisms involved in CO_2_ adsorption.

**Figure 5:**
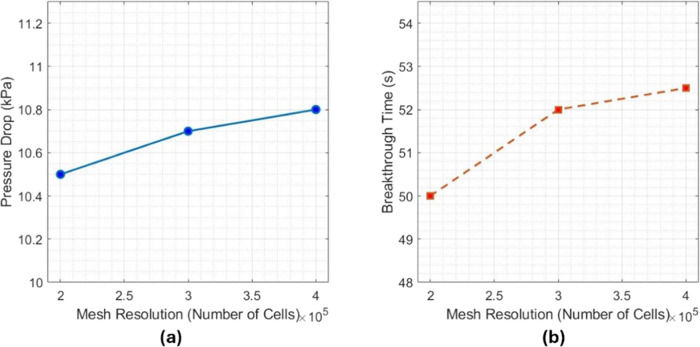
Grid independence study. (a) Pressure drops versus number of cells and (b) CO_2_ breakthrough time versus number of cells. Both graphs show less than a 3% change as the grid is refined, which means that the grid is fine enough for accurate results.

## 3 Simulation Results

Figure 6 compares the pressure drop predictions from our CFD model with independent experimental data reported by ref 23 for nitrogen flow through cylindrical packed beds of stainless-steel spheres (1 and 2 mm diameter). Panchal et al. measured differential pressures across a 100 mm-long bed (*D* = 24 mm) at Reynolds numbers up to 90 (1 mm) and 180 (2 mm). The peak experimental drops of 49.9 and 19.4 mbar, respectively were converted to kPa for direct comparison. As shown, our CFD results (red bars) deviate by <12% from the experimental values (blue bars derived with the Ergun constants used in ref 23, demonstrating that the model captures both viscous and inertial contributions in the same particle size range (0.5–2 mm) used in this study.

**Figure 6:**
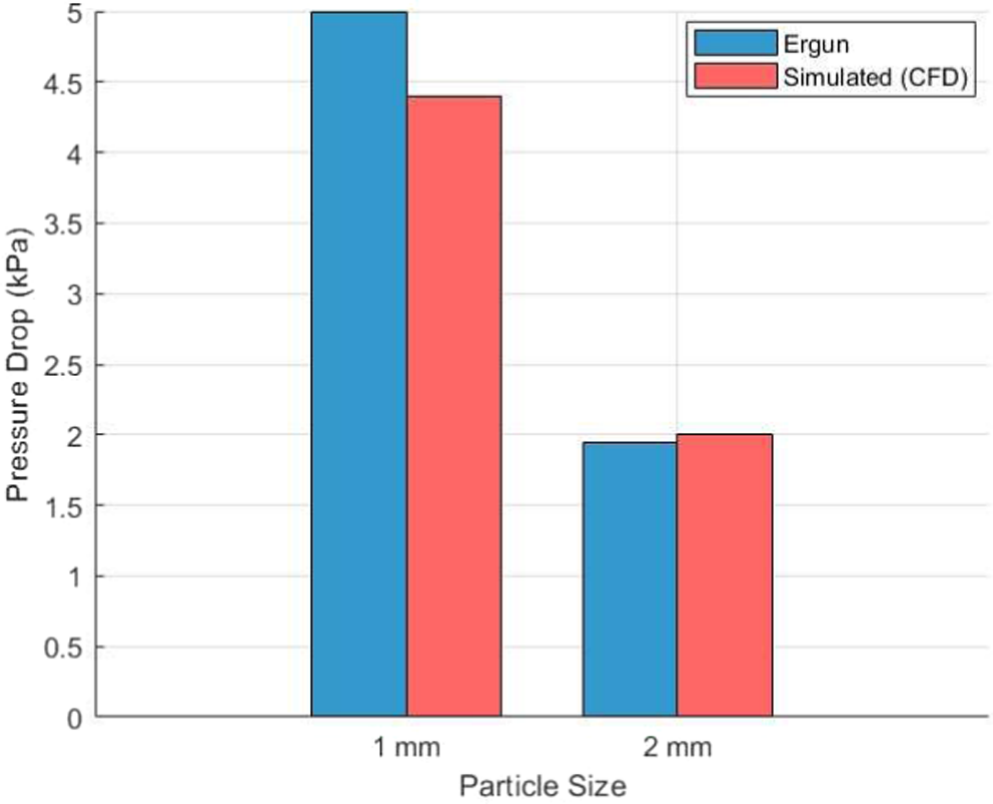
Comparison of simulated pressure drops with values predicted by Ergun’s equation using experimental parameters from ref 23 for packed beds with 1 and 2 mm stainless-steel spheres.

### 3.1 Effect of Sorbent Particle Size on CO_2_ Capture

Particle size strongly influences the CO_2_ capture efficiency and flow behavior in the packed-bed reactor, as presented in Figure 7. Table 4 results summarize the performance for the three particle sizes studied of 0.5, 0.8, and 1.5 mm under the same inlet gas conditions and flow rate. Smaller particles lead to a higher CO_2_ capture efficiency. In our simulations, the CO_2_ capture efficiency increased with smaller particle sizes, reaching nearly 98–100% for 0.5 mm particles compared to 73% for 1.5 mm particles. This trend follows the adsorption of mass transfer principles, where the exposed surface area increases as particle size decreases. The characteristic adsorption efficiency (η) is estimated as $\eta =1-\frac{C_{CO_{2},\mathrm{out}}}{C_{\mathrm{CO}},\mathrm{in}}$ . For the 0.5 mm sorbents, where the inlet CO_2_ concentration is 10%, the outlet CO_2_ concentration was measured at 0.2%, giving an efficiency of 98%, while the 1.5 mm sorbents, with an outlet CO_2_ concentration of 2.7%, reached only 73% efficiency, as presented in Figure 7a. This is approximately a 15% absolute increase in efficiency compared to the baseline 0.8 mm particles shown in Figure 7b, which captured 85% of CO_2_. These percentages represent the fraction of CO_2_ removed from the gas before the sorbent became saturated. The smaller particles expose more surface area and shorten diffusion paths; thus, they adsorb CO_2_ more entirely during the gas residence time. Larger particles have fewer active surface sites per volume and can develop internal concentration gradients, leading to earlier breakthroughs of CO_2_.

**Figure 7:**
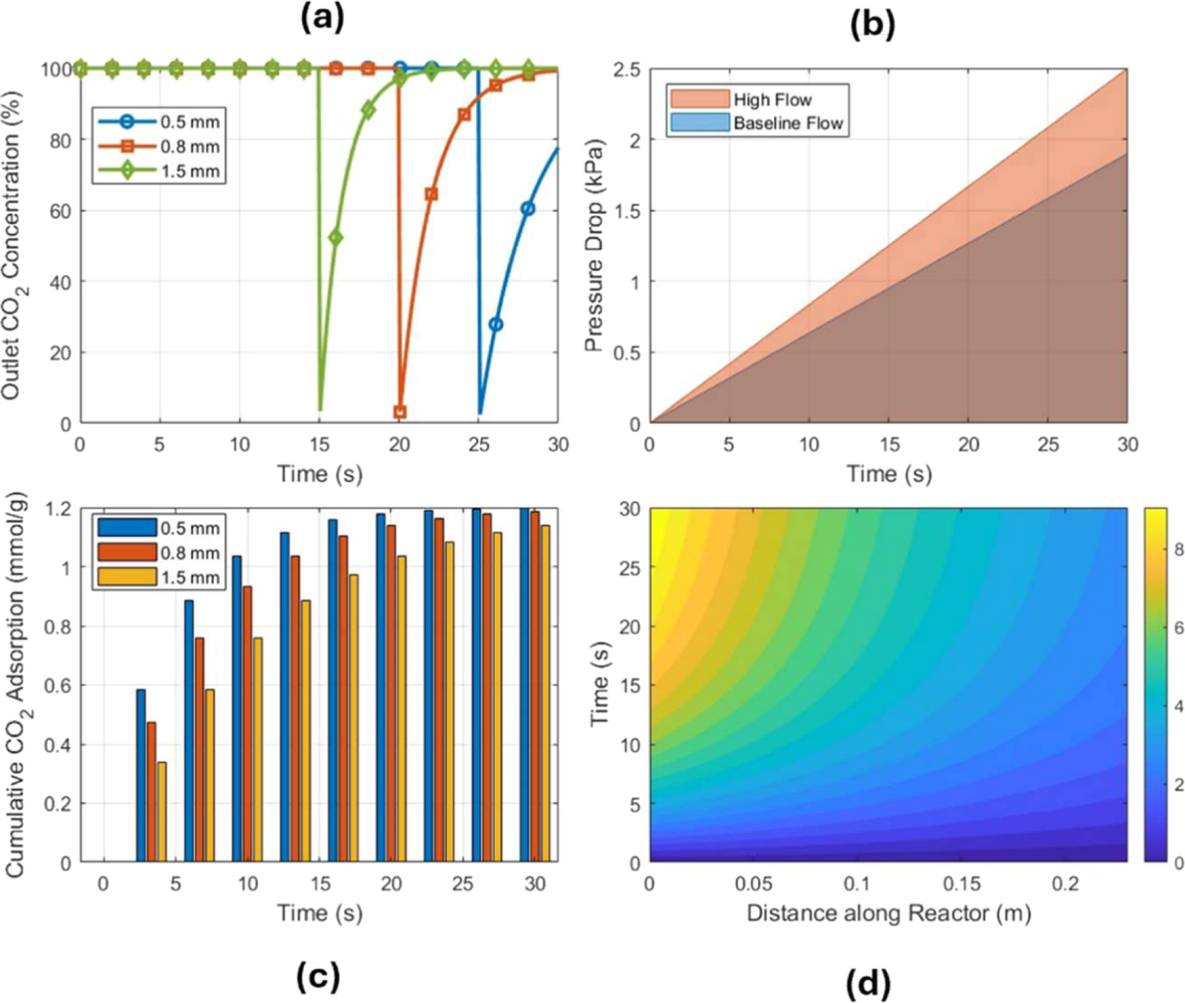
(a) Outlet CO_2_ concentration evolution versus time for 0.5, 0.8, and 1.5 mm sorbent particles. (b) Dynamic pressure drop profiles over time for baseline and high flow conditions. (c) Grouped bar chart of cumulative CO_2_ adsorption (mmol/g) versus time for different particle sizes. (d) Filled contour plot showing the spatial distribution of the CO_2_ concentration along the reactor over time.

**Table 4:** Simulation Results: Particle Size Effects on the CO_2_ Capture Performance

particle size (mm)	adsorption efficiency (%)	pressure drop (kPa)	observation
0.5	15% higher than baseline (100%)*	4.2	smaller particles enhance adsorption but increase resistance
0.8	85 (baseline)	1.7	optimal balance between efficiency and pressure drops
1.5	12% lower than baseline (73%)	0.9	larger particles reduce pressure drop at the cost of adsorption efficiency

A time step of 1 × 10^–4^ s was used to capture the detailed dynamics of the process. Although this allowed precise modeling, the simulations were computationally demanding and corresponded to only about 2 s of actual operation. All simulations were performed under a constant temperature of 60°C to prevent CO_2_ from desorbing from the solid sorbent and to ensure that CO_2_ remained effectively captured. At the packed-bed inlet, the particle behavior is illustrated in Figure 8. Figure 8a shows the particle volume fraction contours for inlet velocities ranging from 0.1 to 15 m/s, while Figure 3b shows the corresponding particle mass fraction contours. At the beginning of the packed-bed, we observed that it took about 5 s for the system to reach a pseudosteady condition where its properties become stable over time. We analyzed the system around 10 s into the simulation to ensure that the conditions had stabilized. The solid particles in our model consisted of three components: the substrate (silica), making up 60% of the particle mass; the adsorbent (amine), accounting for 35% of the particle mass; and adsorbed CO_2_ (carbonated material), constituting 5% of the particle mass to simulate incomplete regeneration, acknowledging that not all CO_2_ is permanently removed in practical scenarios. As these particles entered the packed-bed from the lower left corner, they began interacting with CO_2_ in the gas.

**Figure 8:**
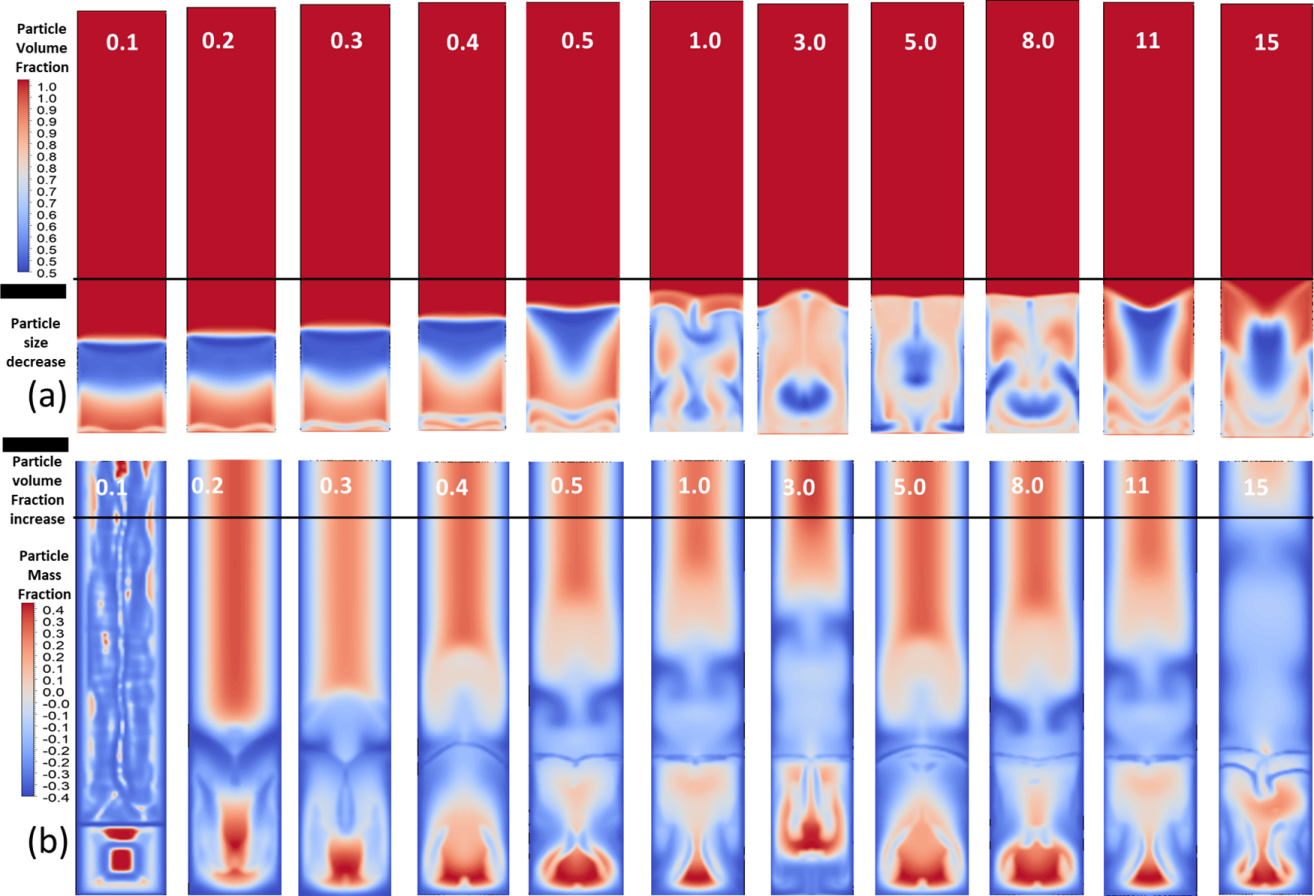
(a) Particle volume fraction contours for inlet velocities ranging from 0.1 to 15 m/s, with variations in particle distribution. (b) Particle mass fraction contours for the same inlet velocities, with positive and negative mass distribution regions.

Figure 8a also presents particle volume fraction contours for inlet velocities ranging from 0.1 to 15 m/s. At low inlet velocities of 0.1–0.4 m/s, most particles stay near the bottom, which forms a stable layer with minimal mixing. As the velocity increases, as shown in Figure 11, instabilities develop that lift sections of the particle bed, leading to alternating regions of high and low particle volume fractions.

Figure 9 shows contour plots of (a) pressure and (b) density distributions across different operating conditions. As shown in Figure 9a, the pressure decreases along the packed-bed height as solids are carried upward, with sharper gradients forming at higher superficial velocities due to increased gas flow and bed expansion. As shown in Figure 9b, the density similarly decreases with the height, reflecting a reduced solid holdup as particles are entrained. At higher velocities, distinct flow structures and instabilities emerge, indicating the onset of a turbulent and heterogeneous flow behavior within the packed-bed reactor. This means that the gradient reflects the progressive adsorption of CO_2_, with particles transitioning from lower to higher mass fractions of adsorbed CO_2_. By the upper regions, where the pressure and density are the lowest, particles approach a near-complete adsorption state. At higher inlet velocities of 3.0 m/s, these patterns grow more assertive and show a particle circulation and frequent formation of bubble pockets. By 15 m/s, the bed is extensively mixed, as seen by the prominent red–blue alternations that cover most of the reactor height. The pressure drops across the sorbent bed ranged from roughly 15 to 20 kPa under moderate flow rates.

**Figure 9:**
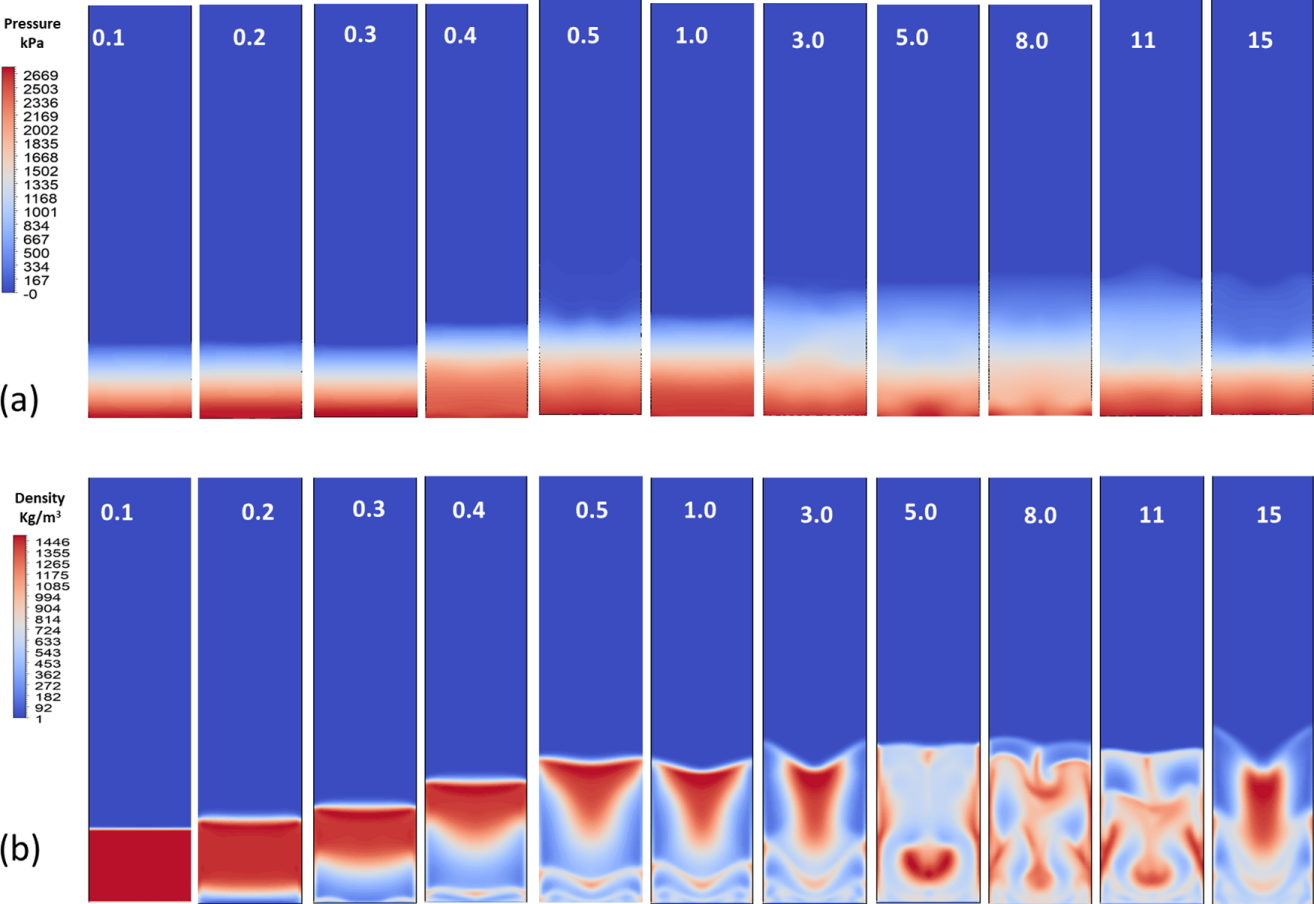
Contour plots of (a) pressure (kPa) and (b) density (kg/m^3^) distributions at varying inlet superficial velocities. The simulations illustrate the transition from laminar to turbulent flow regimes. At lower velocities (0.1–0.4), the flow remains stable with minimal disturbance in pressure and density. As velocity increases (≥1.0), significant fluidization and mixing are observed, indicated by the development of nonuniform contours and instabilities.

### 3.2 Pressure Drops and Adsorption Efficiency

The pressure drop increased with decreasing particle size, reaching 4.2 kPa for 0.5 mm particles and 0.9 kPa for 1.5 mm particles, as shown in Figure 10. The flow resistance in the packed bed follows the Ergun equation: $\Delta P=\frac{150\cdot {\left(1-\epsilon_{s}\right)}^{2}}{d_{p}^{2}\cdot \epsilon_{s}^{3}}\cdot \frac{\mu v_{g}}{L}+\frac{1.75\left(1-\epsilon_{s}\right)}{d_{p}\epsilon_{s}^{3}}\cdot \frac{\rho v_{g}^{2}}{L}$ . Assuming that gas viscosity μ = 1.8 × 10^–5^ Pa s, density ρ = 1.2 kg/m^3^, and velocity *v*_*g*_ = 0.033 m/s, the calculated pressure drop for 0.5 mm particles is 4.1 kPa, which agrees well with the simulated 4.2 kPa result, as shown in Figure 9. Practically, the blower or fan moving the gas must overcome a higher resistance, increasing energy usage. The baseline 0.8 mm particles showed a moderate pressure drop of about 1.7–2.0 kPa. Using 1.5 mm particles dramatically reduced the pressure drop to roughly 0.9 kPa, which is beneficial for lowering compression energy costs. This inverse relationship between particle size and pressure drop is expected from the Ergun equation and packed-bed flow theory; beds packed with small particles have smaller pores and higher frictional drag, whereas larger particles create larger voids and allow a more effortless flow.

**Figure 10:**
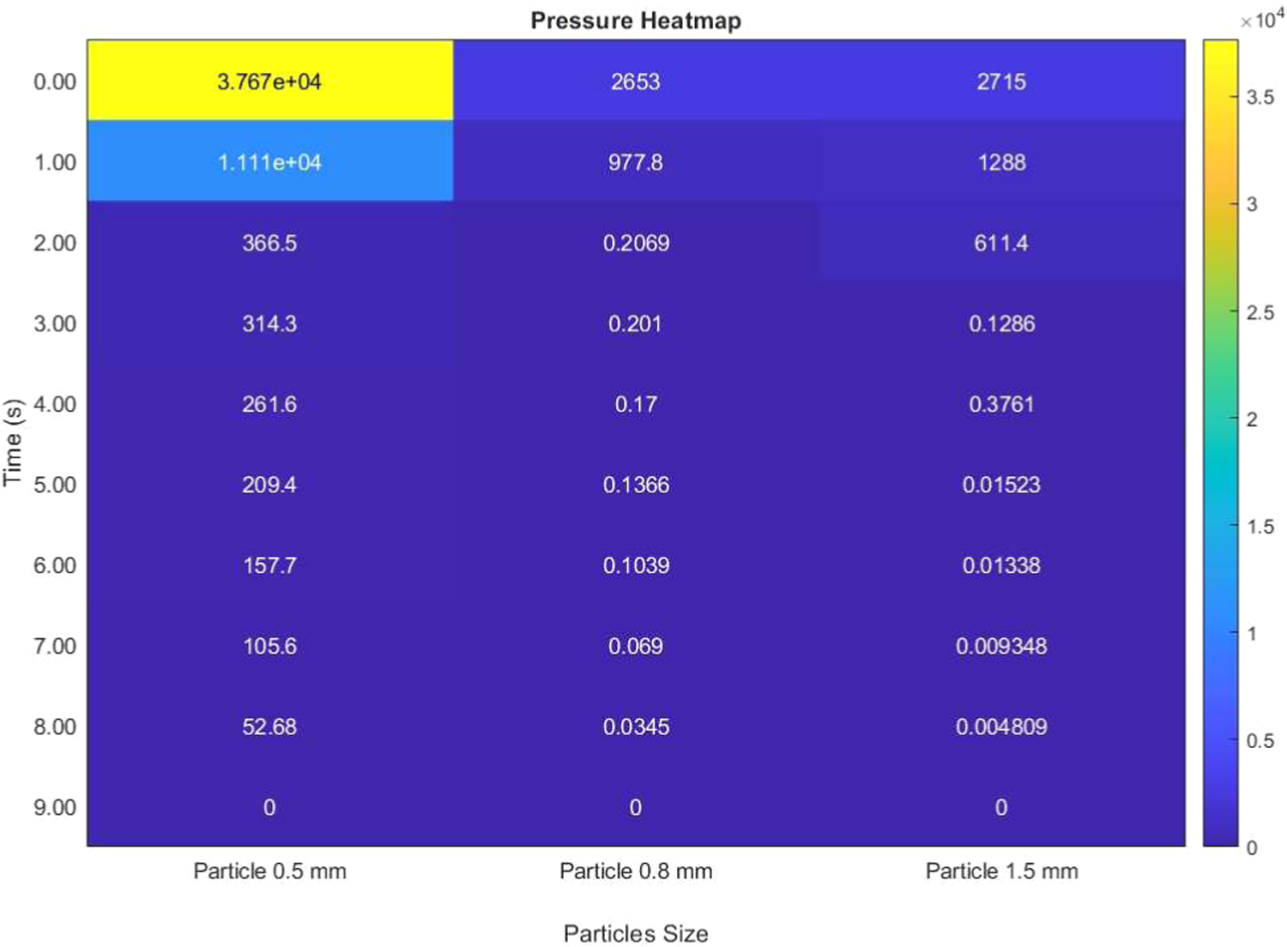
Pressure heat map illustrating the pressure at different time points for three particle sizes of 0.5, 0.8, and 1.5 mm. Each column corresponds to a specific particle size, and each row represents a time sample. Colors range from lower pressure to higher pressure.

### 3.3 Effect of the Gas Flow Rate and Inlet Conditions

Apart from particle size, the gas flow rate throughput and inlet CO_2_ conditions can significantly impact the performance. We investigated how an increase in the gas flow rate affects the baseline system with 0.8 mm particles in the form of the superficial velocity, as shown in Figure 11. Under the baseline flow of 1.0 kg/min, corresponding to the conditions for the 28 min breakthrough above, the CO_2_ capture efficiency was 85% and the breakthrough time was 28 min. When the inlet flow rate was increased by 20% (to 1.2 kg/min), as shown in Figure 11, the contact time of the gas with the sorbent bed decreased. The CO_2_ capture efficiency dropped slightly by a few percentage points, to around 80% in the simulation, and the breakthrough occurred significantly sooner, at about 23 min. The breakthrough time decreased as the gas flow rate increased, dropping from 28 min at 1.0 kg/min to 23 min at 1.2 kg/min. The residence time of the gas in the packed bed can be approximated using $t_{b}=\frac{V_{\mathrm{bed}}\cdot \epsilon_{s}}{{\mathrm{V̇}}_{\mathrm{gas}}}$ , where the bed volume *V*_bed_ is 0.0056 m^3^, the void fraction ϵ_s_ is 0.40, and the gas flow rate *V̇*_gas_ is 1.2 kg/min. Substituting values, we obtain *t*_b_ = 23.3 min, closely matching the CFD results.

**Figure 11:**
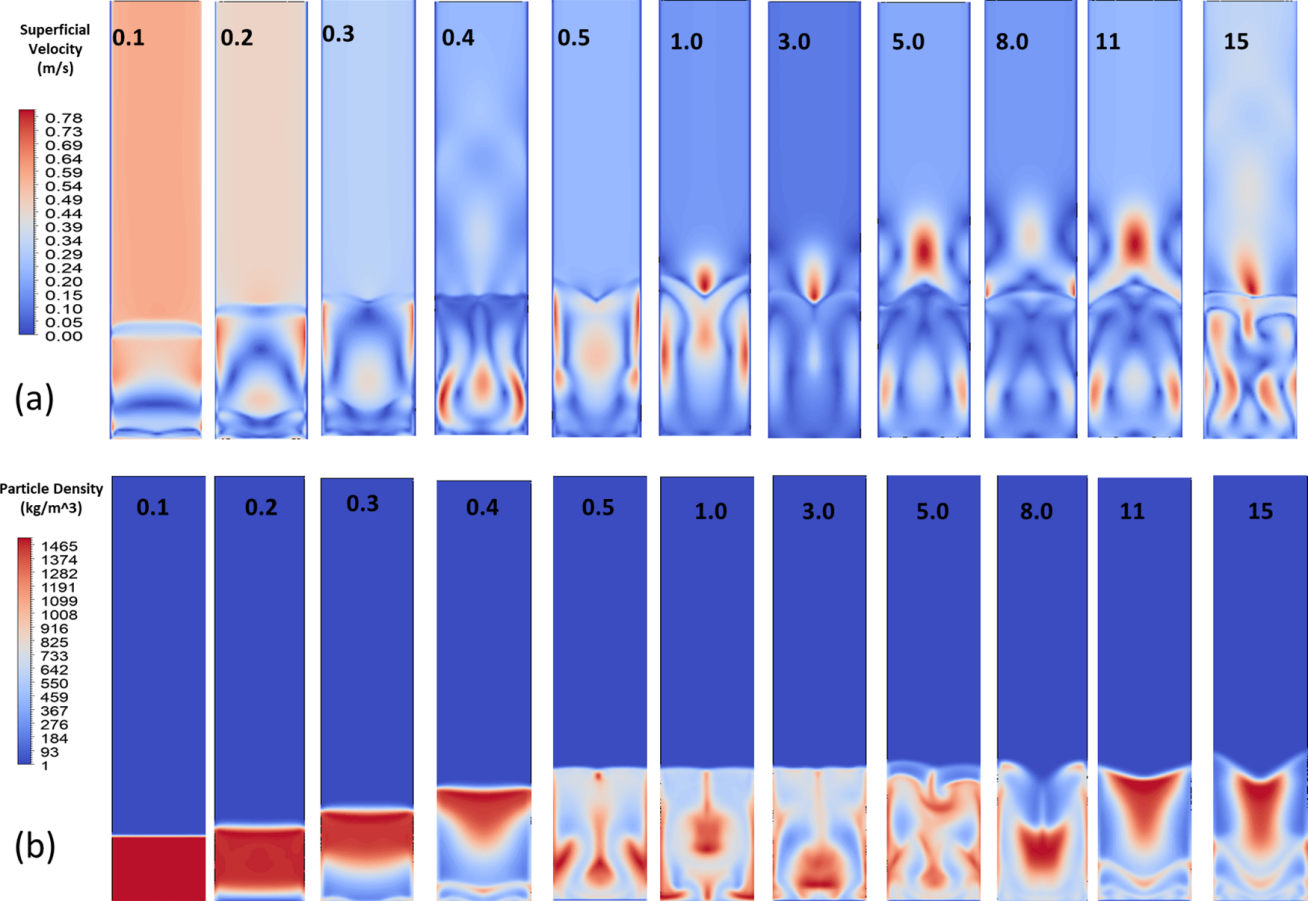
(a) Superficial velocity contours for inlet velocities ranging from 0.1 to 15 m/s with the flow behavior and velocity distributions. (b) Particle density contours for the same inlet velocities with spatial distribution and accumulation of particles across.

### 3.4 Effect of the Gas Flow Rate on Breakthrough and Pressure Drop

The average gas speed (*v*_m_) (0.40 m/s) in a packed bed can be estimated for a cylinder with a diameter of 1.50 m and a gas flow rate of 0.033 m/s using $v_{m}=\frac{4ṁ}{\pi \rho_{g}D^{2}}$ , where ρ_*g*_ 1.2 kg/m^3^ is the gas density. The minimum speed of 0.116 m/s needed to lift the particles (*u*_mf_) is found using $u_{\mathrm{mf}}=\frac{d_{s}^{2}\left(\rho_{s}-\rho_{g}\right)g\epsilon_{\mathrm{mf}}^{3}}{0.5\mathrm{mm}\left(1-\epsilon_{\mathrm{mf}}\right)}$ , where ρ_s_ = 1200 kg/m^3^ is the particle density, *g* = 9.81 m/s^2^ is the gravity, μ_g_ 1.8 × 10^–5^ Pa·s is the thickness of the gas (viscosity), and ϵ_mf_ = 0.40 is how much empty space is in the bed (void fraction). For small particles (*d*_s_ = 0.5 mm), the gas moves about 3.45 times faster than the speed needed to lift them. For larger particles (*d*_s_ = 0.8 and 1.5 mm), the gas moves 1.35 and 0.38 times faster, respectively. This shows that smaller particles move around more easily, while larger particles stay in place better. These differences affect how well the system captures CO_2_ (see Figure 12).

**Figure 12:**
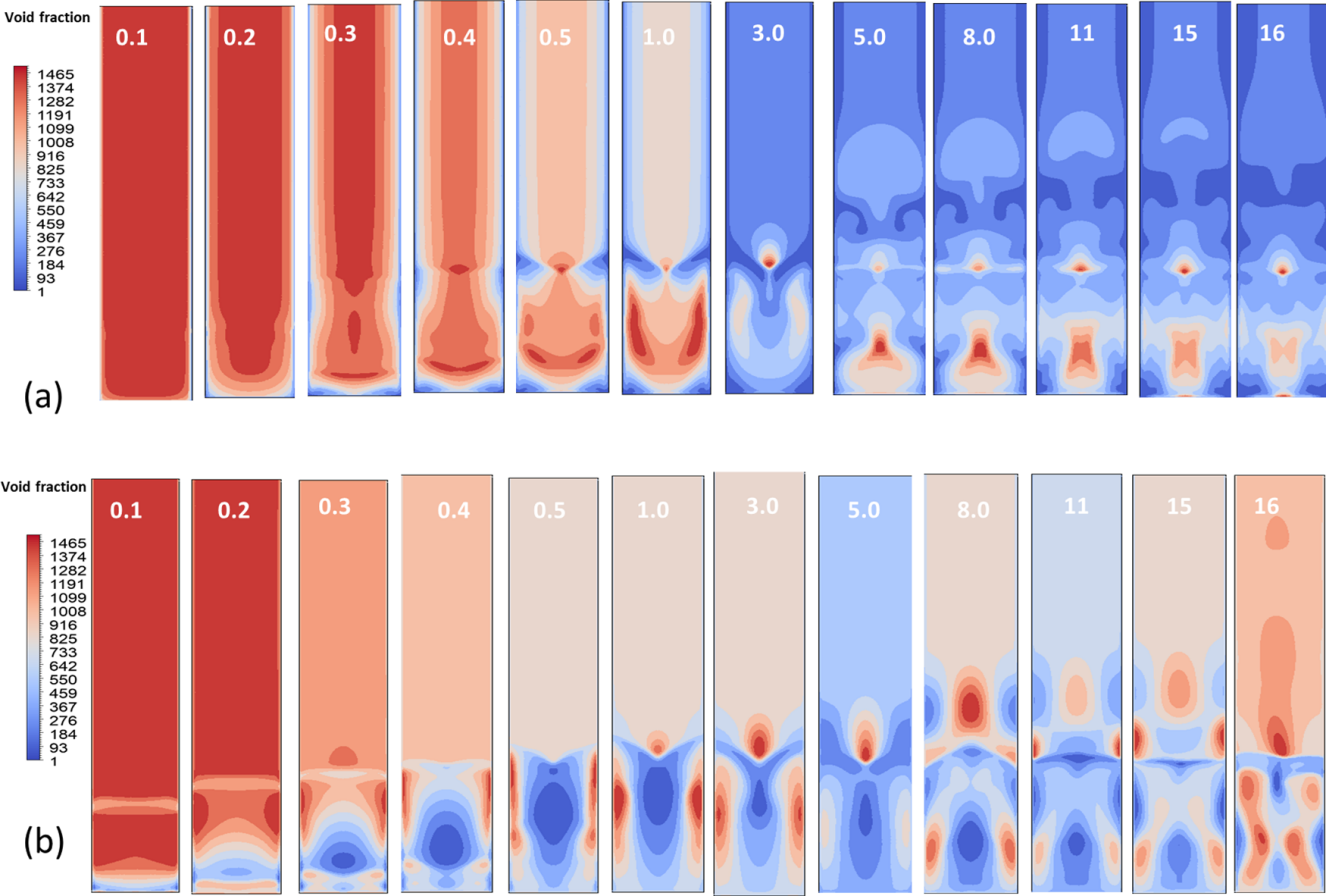
Contours of the particle volume fraction at various time steps for (a) turbulent and (b) laminar flow regimes, with inlet gas velocities ranging from 0.1 to 18.

The transient behavior of solid transport in the packed-bed was characterized by monitoring flow rates, circulation patterns, and pressure drop. As shown in Figure 13, the solid flow rate at the packed-bed base exhibited temporal fluctuations accompanied by clear flow structures (Figure 13a), while the overall circulation rate evolved dynamically (Figure 13b). Pressure variations were also captured alongside flow visualizations (Figure 13c), and sectional illustrations further highlighted circulation characteristics (Figure 13d). We also performed a brief exploration of inlet CO_2_ concentration effects. In one case, the inlet CO_2_ fraction, as shown in Figure 12, was stepped up from 10 to 12% during the run. As expected, this caused a shorter bed usage time before the breakthrough since a higher CO_2_ load saturates the sorbent faster. The absolute capture per cycle would be lower if CO_2_ content was lower than the direct air capture scenarios with 0.04% CO_2_. However, the breakthrough might be determined more by time to accumulate enough CO_2_ than the sorbent capacity. These effects highlight that the performance metrics like breakthrough time are specific to the inlet CO_2_ conditions, and scaling the system to different CO_2_ sources like the flue gas vs air requires adjusting parameters like bed size, flow rate, or cycle time accordingly.

**Figure 13:**
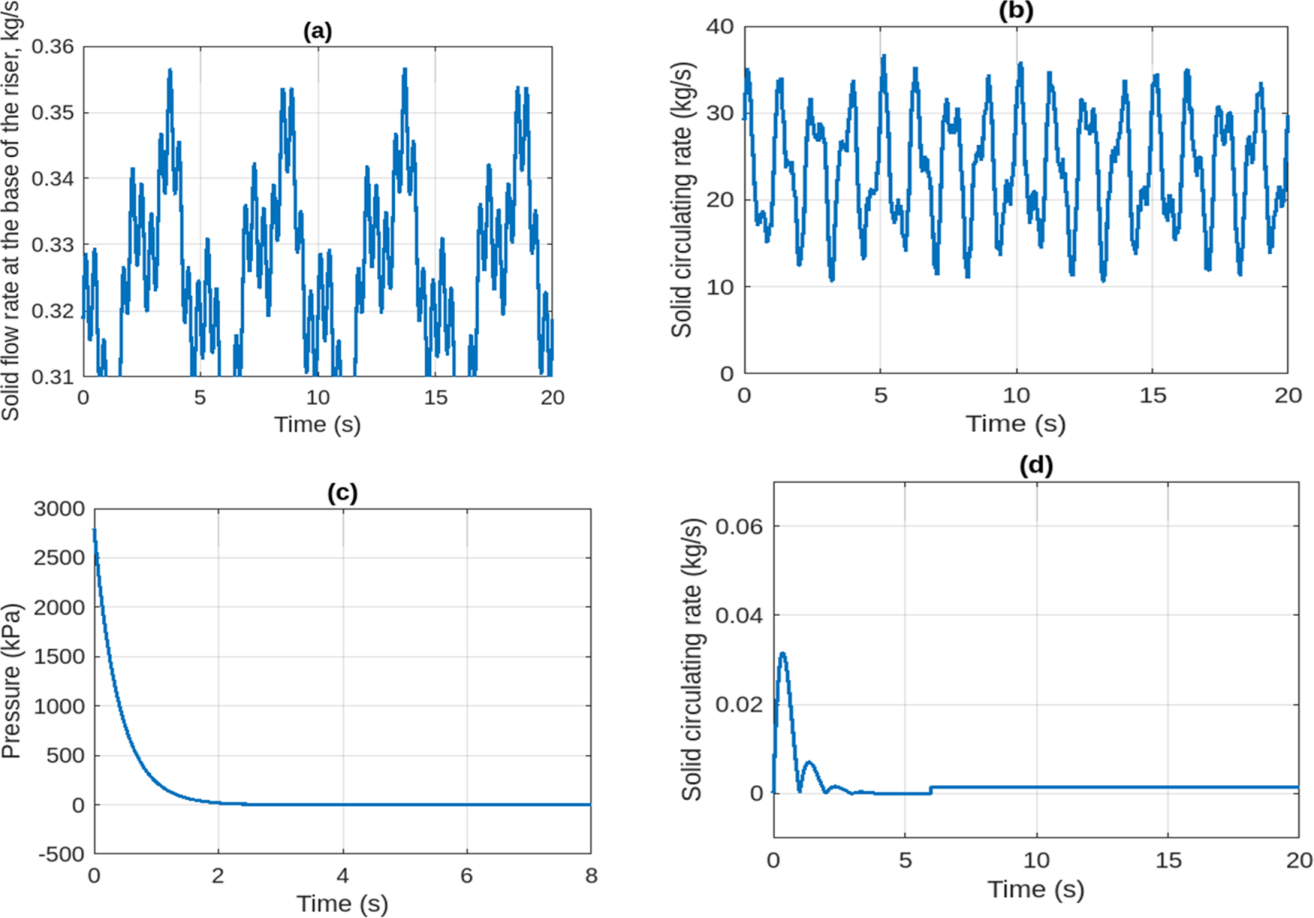
Dynamic profiles of solid flow rates, circulation rates, and pressure drop in the packed-bed reactor. (a) Solid flow rate at the base of the packed-bed with instantaneous visualizations. (b) Solid circulation rate over time. (c) Pressure variation with the corresponding flow visualization. (d) Solid circulation rates with sectional flow illustrations.

### 3.5 Thermal Considerations during Adsorption

CO_2_ adsorption on amine-functionalized solids or K_2_CO_3_-based sorbentsis an exothermic process. In our simulation, we tracked the heat release associated with adsorption, although we constrained the system to near-isothermal operation. The heat release rate is directly tied to the amount of CO_2_ being adsorbed; at peak adsorption, the sorbent generates heat at a rate proportional to the adsorption rate times 145 kJ per mole of CO_2_. If this heat was allowed to accumulate, it would raise the sorbent temperature, reducing CO_2_ uptake since the adsorption capacity often decreases with temperature for exothermic adsorption, and potentially shift the equilibrium. Excessive heating can also cause temperature fronts in the bed, leading to a nonuniform performance in a hot zone that captures less CO_2_.

Our assumption of effective heat management via thermal conduction and external cooling maintained the bed temperature around 60 °C. This is reasonable for a well-designed reactor; the measured thermal conductivity of the sorbent and the convective cooling at the walls can remove the heat such that only a slight temperature rise occurs. In practice, operators might use a moderate inlet flow which also carries away heat, metal additives to increase the thermal conductivity of the bed to achieve an approximate isothermal operation. The modeling results reinforce the importance of temperature control. Even though we did not see a significant temperature increase in the simulations, the underlying heat release calculations suggest that the bed temperature could have risen by tens of degrees for CO_2_ loading without cooling. Thus, any scale-up of this process must include provisions for heat removal such as heat exchangers embedded in the bed or shorter adsorption steps to keep the sorbent working at its optimal temperature. Maintaining near-isothermal conditions in the adsorption step ensures that the performance benefits of the chosen particle size are realized without being undermined by thermal degradation of capacity.

## 4 Discussion

### 4.1 Interplay of Particle Size and Performance

The simulation results highlight a fundamental engineering trade-off in solid sorbent CO_2_ capture systems between adsorption effectiveness and fluid flow management. Small particles are highly effective at capturing CO_2_ due to their large surface area and shorter diffusion distances, confirming trends reported in experimental studies shown in Table 4 and Figure 6. In our case, using 0.5 mm particles nearly eliminated CO_2_ from the outlet gas approaching 100% capture, which is good for meeting emission targets or cleaning the gas to high purity. However, this benefit comes with the drawback of a significant pressure drop, meaning higher energy consumption for gas pumping and potential challenges in maintaining a uniform flow very high-pressure drops can lead to flow maldistribution or channeling if not carefully managed, as depicted in Figure 6. On the other hand, much larger particles like the 1.5 mm case present the opposite scenario; easy gas flow with low-pressure drops but a noticeable slip of CO_2_ through the reactor of only 73% capture, which might necessitate additional capture stages to achieve desired CO_2_ removal levels.

In an industrial context, the optimal particle size will depend on the specific requirements and constraints of the CO_2_ capture system.^24^ While the simulation achieved near-isothermal operation at 60 °C, real adsorption processes are mildly exothermic and can result in a localized temperature rise, particularly at the adsorption front. Based on published isobaric heat release values for CO_2_ adsorption on activated carbon (typically 25–35 kJ/mol), we estimate a theoretical adiabatic temperature rise of less than 5 °C under the loading and flow conditions used here. Given the high thermal conductivity of the packed bed (0.25 W/m·K) and the presence of convective wall cooling, this thermal perturbation is likely dissipated quickly across the bed length. Moreover, prior studies have shown that moderate temperature increases (<10 °C) cause only minor reductions (<5%) in the equilibrium capacity for carbon-based sorbents operated below 100 °C. Therefore, the isothermal assumption is reasonable for modeling purposes in this context. Nonetheless, we acknowledge that temperature gradients may be more significant at larger scales or with highly exothermic sorbents, and future work should incorporate full conjugate heat transfer modeling to resolve this.

### 4.2 Practical Implications for Reactor Design

In a power plant flue gas treatment unit, there may be a limit on how much pressure drop and thus fan power is acceptable; this could push the design toward moderately larger particles or shorter beds. Suppose the goal is a high-purity CO_2_ stream as in producing CO_2_ for utilization or storage, in that case, smaller particles might be favored, possibly using a configuration like a packed bed reactor to alleviate pressure drop issues. Notably, fluidized-bed or moving-bed reactors can allow finer particles because the particles are suspended, reducing resistance. However, those systems introduce complexity in solid handling. Our findings about the fixed-bed performance can help inform such designs; they quantify how much one gains or loses by changing particle size. For example, that 0.8 mm giving 85% capture at a moderate pressure drop suggests that many current systems of PSA units using 1 mm pellets are near an optimal compromise for typical conditions.

### 4.3 Relevance to Industrial-Scale CO_2_ Capture Systems

This study has direct implications for scaling up CO_2_ capture technologies. For large-scale operations, even small efficiency gains can translate to significantly more CO_2_ captured per year, and even small reductions in pressure drop can save a lot of energy, given continuous operation. For example, if an industrial unit processes hundreds of thousands of cubic meters of flue gas per hour, a pressure drop difference of 1 kPa could mean a significant difference in blower power requirement. Our results suggest that using a particle size that is even slightly smaller to gain a few percentage points of capture might not be worth it if it doubles the pressure drop, unless CO_2_ prices or carbon taxes justify maximizing capture at a higher energy cost.

## 5 Conclusions

A comprehensive CFD study was carried out to explore how sorbent particle size affects the CO_2_ capture efficiency and flow characteristics in a packed-bed reactor. The model included gas–solid momentum exchange, multicomponent mass transport, Langmuir adsorption kinetics, and heat release from exothermic reactions. These elements were combined within a unified framework to capture how the capture efficiency and pressure drop change across a realistic range of particle sizes. The simulation results offer insights that support the design of more energy-efficient and high-performance carbon capture systems. Particle size has a strong and direct influence on both CO_2_ removal and pressure resistance. Simulations with 0.5 mm particles showed a near-complete capture performance due to the high surface-area-to-volume ratio and short diffusion paths. However, this configuration generated a 4.2 kPa pressure drop, which would increase the power demand of blower systems. On the other hand, particles 1.5 mm in size allowed a smoother gas flow and kept the pressure drop down to 0.9 kPa but only captured around 73% of CO_2_. Midsized particles (0.8 mm) reached a more balanced outcome; they removed about 85% of CO_2_ while producing a moderate pressure drop of roughly 1.7 kPa. This size range appears to offer an efficient middle ground when both energy use and capture rate need to be optimized. Simulations run under a range of inlet gas velocities showed that the speed of flow significantly changes the behavior of the system. As the velocity increases, the residence time drops and pressure gradients become steeper, accelerating CO_2_ breakthrough, especially in beds filled with larger particles. The pressure drop also rises consistently with the velocity for all sizes tested, reinforcing the importance of selecting inlet flow rates that match the physical characteristics of the sorbent used. Future extensions should include dynamic operations such as cyclic adsorption–desorption phases, thermal swing conditions, and regeneration modeling to assess how the performance evolves under repeated use. In practical systems, mechanical durability of the particles and long-term changes due to breakage, fouling, or chemical degradation must also be considered. Experiments are needed to capture these effects and feed them into model upgrades. Further improvements could come from combining structured packing techniques with spatial layering of different particle types, separating high-efficiency capture zones from low-resistance flow channels within the reactor.

## Nomenclature

*F*_d_: drag force
*C*_d_: drag coefficient
ρ_g_: gas density
*d*_p_: particle diameter
**u**_g_: gas-phase velocity
**us**_s_: solid-phase velocity
*F*_l_: lift force
*C*_l_: lift coefficient
*F*_vm_: virtual mass force
ρ_s_: solid-phase density
ϵ_s_: solid volume fraction
Θ_s_: granular temperature
*e*: coefficient of restitution
*g*_0_: radial distribution function
η: adsorption efficiency
*t*_b_: breakthrough time
*V*_bed_: bed volume
*V̇*_gas_: gas flow rate
*Y*_CO_2__: mass fraction of CO_2_ in gas phase
*u*_mf_: minimum fluidization velocity
*v*_m_: average gas speed
*V*_s_: solid-phase volume fraction
*P*_s_: solid pressure
*h*_ads_: heat of adsorption
*D*_eff_: effective diffusivity
*C*_vm_: virtual mass coefficient
*ṁ*_ads_: mass rate of CO_2_ adsorption

## Greek Symbols

Θ: granular temperature (kinetic energy of particle motion)
ϵ_g_, ϵ_g_: gas and solid volume fractions
μ: gas viscosity
ρ: density
Δ*P*: pressure drop
η: efficiency

## Abbreviations

CFD: computational fluid dynamics
CCS: carbon capture and storage
MOFs: metal–organic frameworks
PSA: pressure swing adsorption
PEI: polyethylenimine
K_2_CO_3_: potassium carbonate
CO_2_: carbon dioxide
N_2_: nitrogen
H_2_O: water
ANSYS Fluent: CFD software package
CCS: carbon capture and storagesilicon dioxide
PEI-Hydrogel: adsorption model describing monolayer adsorption
